# Nanoconfined Metal Halide Perovskite Crystallization within Removable Polymer Scaffolds

**DOI:** 10.1021/acs.cgd.5c00073

**Published:** 2025-04-14

**Authors:** Mia Klopfenstein, Lance Emry, Pulkita Jain, Aida Alaei, Ben Schmelmer, Andrew Chou, Trinanjana Mandal, Min-Woo Kim, Eray S. Aydil, Tsengming Chou, Stephanie S. Lee

**Affiliations:** † Molecular Design Institute, Department of Chemistry, New York University, New York, New York 10003, United States; ‡ Department of Chemical and Biomolecular Engineering, Tandon School of Engineering, New York University, Brooklyn, New York 11201, United States; § Department of Chemistry, New York University, New York, New York 10003, United States; ∥ Department of Semiconductor Engineering, Myongji University, Cheoin-gu, Yongin-si, Gyeonggi-do 17058, Korea; ⊥ Department of Chemical Engineering and Materials Science, Stevens Institute of Technology, Hoboken, New Jersey 07030, United States

## Abstract

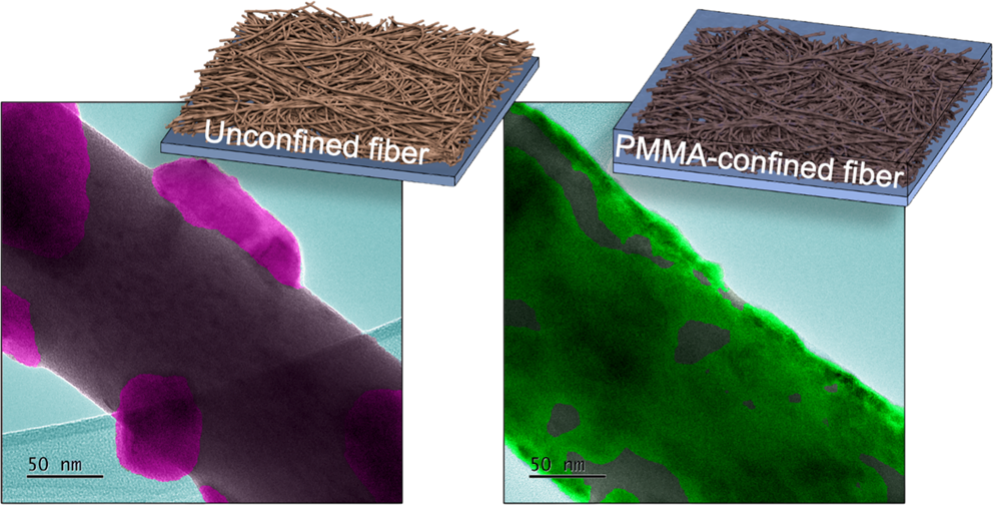

Nanoconfining crystallization to access metastable polymorphs and prescribe crystal orientations typically involves filling inert nanoporous scaffolds with target compounds, resulting in isolated nanocrystals. Such crystal-scaffold composites are unsuitable for optoelectronic devices that require interconnected crystalline pathways for charge transport. Here, we reverse the order of fabricating crystal-scaffold composites by first electrospinning interconnected networks of amorphous methylammonium lead iodide (MAPbI_3_) precursor nanofibers, then introducing a poly(methyl methacrylate) (PMMA) scaffold by spin coating from an antisolvent for MAPbI_3_. PMMA suppresses MAPbI_3_ crystal blooming from the fiber surface during thermal annealing, instead promoting the formation of densely packed polycrystalline networks of MAPbI_3_ crystals at the fiber/PMMA interface. Near-IR photodetectors comprising densely packed MAPbI_3_ nanocrystals grown within a PMMA scaffold in a coplanar electrode geometry exhibit photocurrents up to 60 times larger than those comprising fibers annealed without PMMA. These results indicate that MAPbI_3_ crystals form a percolated network for charge carriers to flow through PMMA-confined fibers, resulting in significantly improved photodetector performance.

## Abstract 2

False-colored TEM images of electrospun MAPbI_3_ fibers highlighting MAPbI_3_ crystals that formed during thermal annealing in the absence and presence of a confining polymer scaffold. Large, isolated crystals bloomed from the surfaces of unconfined fibers, while confined fibers exhibited small, interconnected crystals at the fiber surface.

## Introduction

Crystals grown in nanoconfined spaces deviate from those grown in the bulk. Many early studies in the field characterized melting and glass transition point depressions with decreasing crystal size following the Gibbs–Thomson equation.^1−4^ In a series of papers beginning in 2004,^5−16^ Ward established nanoconfinement to alter the relative thermodynamic stabilities of polymorphs in molecular compounds. As the surface energy contribution to the Gibbs free energy becomes increasingly significant with decreasing crystallite size, relative polymorph stabilities can switch at a given temperature and pressure.^5,17,18^ Nanoconfined glycine crystals, for example, were discovered to exist in the β-phase,^14^ stable indefinitely against transformation to the thermodynamically favored α-phase in the bulk.^9^ First using partially etched block copolymer monoliths^11−16^ and later anodized aluminum oxide (AAO)^9^ as scaffolds with uniaxially aligned crystallization chambers, Ward further demonstrated that molecular crystals preferentially orient with their fast growth direction aligned with the long axes of nanopores.^19^

These seminal discoveries by the Ward group laid the groundwork to use nanoconfinement as a strategy to optimize semiconductor crystals for optoelectronics.^20,21^ Metal halide perovskites, for example, undergo rapid polymorph conversion^22^ and degradation in moisture and air.^23,24^ Recent studies have demonstrated that nanoconfinement shifts phase transition thermodynamics and kinetics, often improving stability and optoelectronic properties. Nonperovskite, insulating δ-CsPbI_3_ is thermodynamically favored at room temperature in the bulk, but CsPbI_3_ crystals embedded in cylindrical AAO nanopores can persist in the photoactive γ-phase indefinitely.^25,26^ In AAO nanopores <40 nm in diameter, nanoconfined CsPbI_3_ undergoes triaxial tensile strain which in turn affects the bandgap.^27^ Likewise, the MAPbI_3_ tetragonal β-phase to cubic α-phase transition temperature shifts from 330 K for bulk crystals to 170 K for nanoconfined crystals.^28^

Embedding metal halide perovskites within nanoconfining scaffolds renders them more stable but often impedes optoelectronic processes. When insulating scaffolds, such as AAO, are used, crystal interfaces available for photophysical processes are restricted to the scaffold surface, limiting the incorporation of nanoconfined crystals for optoelectronics. Crystals within nanopores are also isolated from one another, preventing charge transport in all directions except along the nanopore long axis. We recently used nanoporous, semiconducting titanium dioxide scaffolds to direct the crystallization of formamidinium lead iodide (FAPbI_3_) into unconfined vertical nanowire arrays.^29^ Using a two-step deposition method to convert PbI_2_ crystals to α-FAPbI_3_, crystals were vertically oriented within the TiO_2_ scaffold and grew above the scaffold to achieve large surface areas for efficient solar energy harvesting. These α-FAPbI_3_ nanowire arrays exhibited maximum short-circuit current densities of 22–23 mA/cm^2^ due to the large available surface area of the perovskite phase. While nanowire arrays have large surface areas for optoelectronic processes, this method still requires batch-to-batch fabrication. To improve scalability, here we propose a nanoconfinement method that takes advantage of continuous deposition via electrospinning of interconnected fibers.

Here, we achieve nanoconfinement of polycrystalline MAPbI_3_ fibers while maintaining a percolated charge transport network by flipping the order of fabricating nanoconfined crystal-scaffold composites. Specifically, we first electrospun amorphous MAPbI_3_ precursor nanofibers (∼200 nm diameter), then infiltrated the fiber films with poly(methyl methacrylate) (PMMA) as a temporary confining scaffold during thermal annealing to form the crystalline phase. PMMA was subsequently removed by selective dissolution in chlorobenzene, an antisolvent for MAPbI_3_, to reveal the crystalline nanostructure of the fibers. We discovered that PMMA suppressed MAPbI_3_ diffusion and crystal blooming at the fiber surfaces during thermal annealing. As revealed by transmission electron microscopy, MAPbI_3_ crystallization was instead forced to proceed along the fiber surfaces to form smaller, more densely packed nanocrystals. The extent of percolation between MAPbI_3_ crystals was probed by fabricating photodetectors with a coplanar electrode geometry. Photodetectors comprising PMMA-confined MAPbI_3_ active layers exhibited sixty-fold larger photocurrents and external quantum efficiencies compared to those comprising unconfined MAPbI_3_ fibers, indicating the presence of a PMMA scaffold improves interconnectivity between MAPbI_3_ crystals.

## Experimental Methods

### Preparation of Electrospun Fiber Films

A 0.824 M MAPbI_3_ with 12 wt % PVP (average *M*_w_ = ∼1,300,000 kDa by light scattering) precursor solution was made by dissolving 0.131 g of MAI, 0.380 g of PbI_2_, 0.070 g of PVP dissolved in 1 mL of DMF and stirred at 45 °C for 12 h to obtain a viscous transparent yellow solution. The MAPbI_3_ precursor solution was electrospun onto a grounded aluminum foil plate at a rate of 4 μL/min for 12 min using a syringe pump. A voltage of 17 kV was applied between the syringe tip and aluminum foil collector, which were placed at a distance of 13 cm. For nanoconfined films, electrospinning was followed by spin coating 120 mg/mL PMMA (average *M*_w_ = ∼350,000 kDa by gel permeation chromatography) solution in toluene at 2500 rpm for 30 s directly onto the fiber film. Films were then annealed at 100 °C between 20 and 60 min on a hot plate. All steps were performed in a N_2_-filled glovebox. All chemicals were purchased from Sigma-Aldrich.

### Film Characterization

X-ray diffraction patterns of the films were collected using a Bruker AXS D8 Discover GADDS microdiffractometer with Cu Kα source in reflection mode. The samples were oscillated in two orthogonal directions to average the data over a 1 × 1 mm^2^ area. The film morphology was characterized using a high-resolution scanning electron microscope operated at 3 kV and 100 pA (Oxford Instruments Merlin Carl Zeiss HR-SEM). Energy-dispersive X-ray spectroscopy (EDS) was performed using an Ultim Max detector in the HR-SEM operated at 15 kV and 2 nA. The films were sputter coated with 10 nm of carbon prior to EDS characterization. Absorption and photoluminescence (PL) spectra were collected using a UV–vis–NIR 508 PV Microscope Spectrophotometer (CRAIC Technologies). PL spectra were collected at λ_ex_ = 546 nm. Time-resolved photoluminescence (TRPL) measurements were performed using the time-correlated single photon counting (TSCPC) with a Horiba Delta Diode system. TRPL was excited using a pulsed diode laser at 368 nm with a repetition rate of 1 MHz and detected using a red-enhanced Hamamatsu photomultiplier tube (S-R13456185-980 nm). Emissions at 767 and 762 nm were monitored for unconfined and PMMA-confined films, respectively. Transmission electron microscopy (TEM) was performed with a JEOL 2100Plus TEM at an acceleration voltage of 200 kV.

### Photodetector Fabrication

Glass substrates were sequentially sonicated for 15 min in acetone, methanol, and deionized water. 10 nm of Cr and 100 nm of Au were thermally evaporated at 0.1 and 0.15 nm s^–1^ onto the cleaned glass substrates through a shadow mask to define coplanar electrodes (active area = 0.005 cm^2^). MAPbI_3_ fibers were electrospun directly onto the electrode/glass substrates in a N_2_-filled glovebox following the same recipe previously described. The PMMA-confined film followed a subsequent spin coating of 120 mg/mL of PMMA dissolved in toluene at 2500 rpm for 30 s. Devices were then annealed at 100 °C for 60 min.

## Results and Discussion

**Scheme 1:**
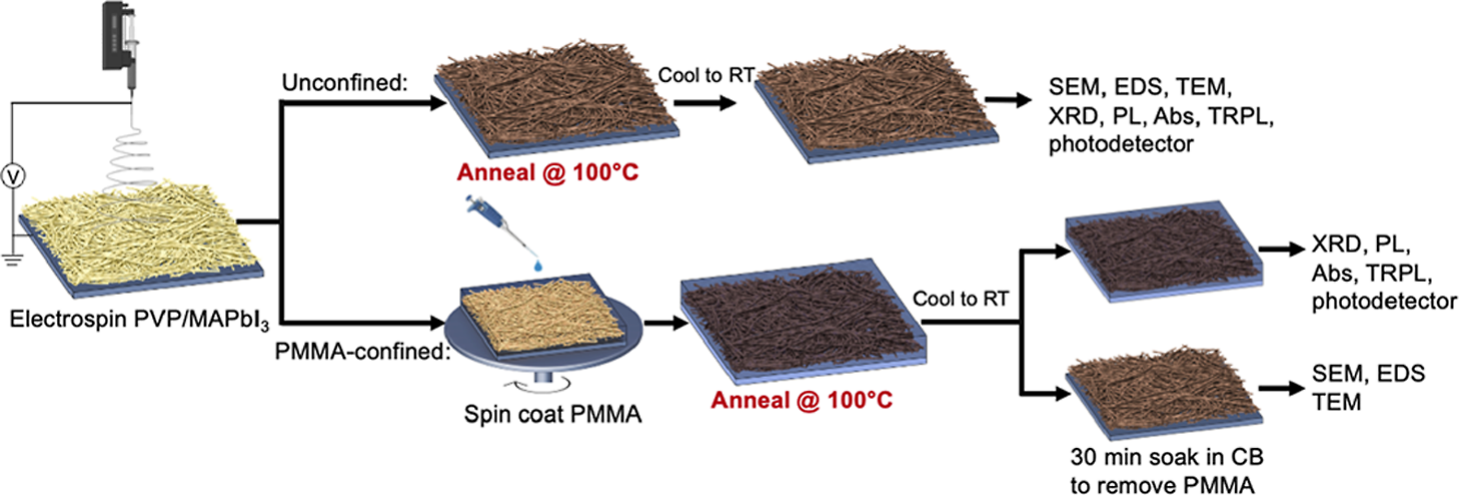
Schematic to Fabricate Unconfined (Top) and PMMA-Confined (Bottom) Films and the Subsequent Characterization for Each Film SEM = Scanning electron microscopy, EDS = Energy dispersive X-ray spectroscopy, TEM = transmission electron microscopy, XRD = X-ray diffraction, PL = photoluminescence spectroscopy, Abs = absorption spectroscopy, TRPL = time-resolved photoluminescence spectroscopy, CB = chlorobenzene, RT = room temperature.

**Figure 1:**
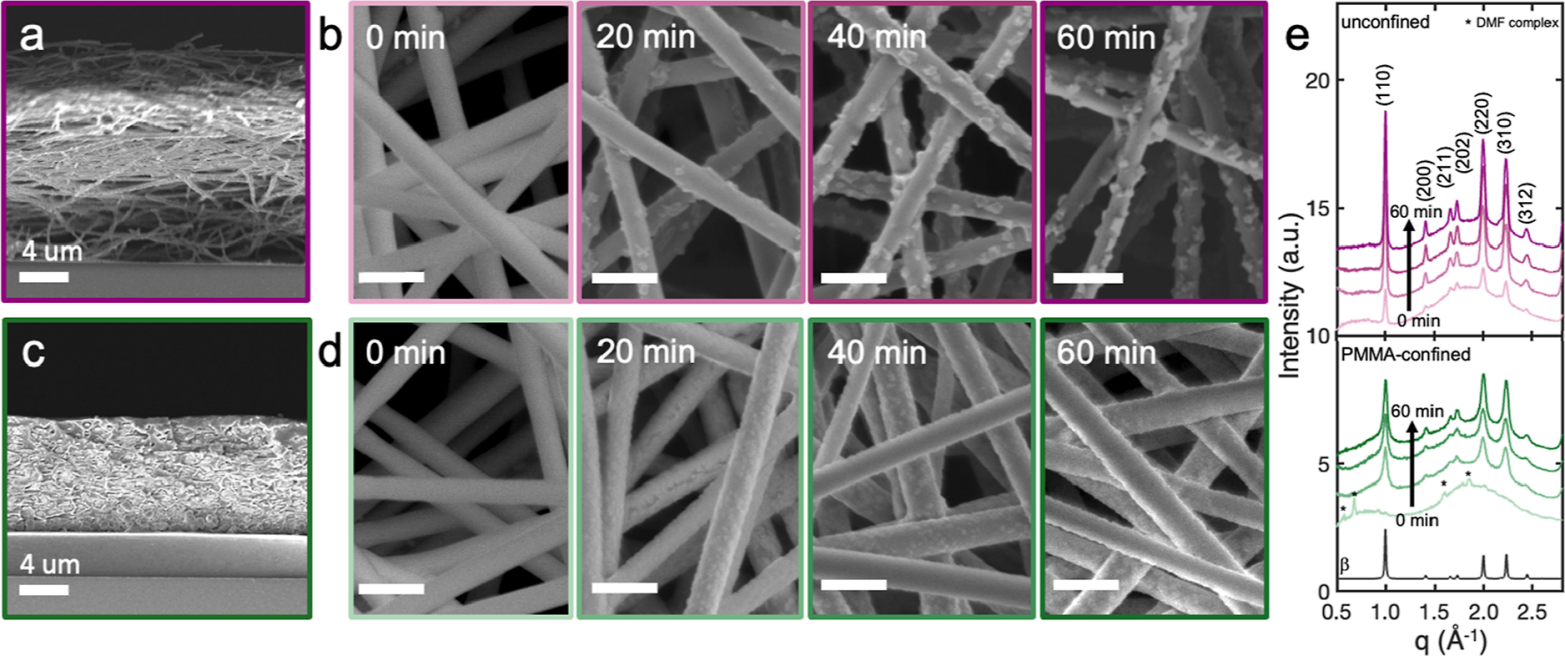
(a) Cross-sectional and (b) top-view SEM images of unconfined fiber films annealed for 60 min and 0, 20, 40, and 60 min at 100 °C, respectively. (c,d) Corresponding images of PMMA-confined samples. PMMA was removed to collect top-view SEM images in (d). Scale bars = 500 nm in (b,d). (e) X-ray diffraction patterns of unconfined (top) and PMMA-confined (bottom) films for different annealing times. Peaks corresponding to a solvent complex phase, (MA)_2_(DMF)Pb_3_I_8_, are indicated by asterisks. Simulated powder pattern of β-MAPbI_3_ is provided in black for comparison.

Solutions containing 88 wt % (69 vol %) MAPbI_3_ precursors and 12 wt % (31 vol %) polyvinylpyrrolidone (PVP), a hydrophilic polymer to add viscosity and elasticity to the precursor solution, were electrospun onto substrates and then thermally annealed either in the absence (Scheme 1, top) or presence of PMMA (Scheme 1, bottom) to form unconfined and PMMA-confined samples, respectively. Immediately after electrospinning MAPbI_3_ precursor solutions, the films appeared pale yellow (Scheme 1), indicating MAPbI_3_ crystals did not form during electrospinning. Cross-sectional (Figure 1a) and top-view SEM images (Figure 1b) of unconfined MAPbI_3_ fibers revealed the presence of a ∼17 μm-thick film after 12 min of electrospinning, comprising smooth, 200 nm-diameter fibers. The films turned dark brown after annealing at 100 °C for 20 to 60 min in a N_2_-filled glovebox. This color change, consistent with MAPbI_3_ formation,^30^ was accompanied by the appearance of isolated crystals protruding from the fiber surfaces in SEM images that increased in number and size with longer annealing times (Figure 1b). The average size of the crystallites was ∼70 nm after annealing for 60 min. This morphology has been reported for other electrospun perovskite fibers^31−33^ as a result of crystal blooming during phase separation of the small molecule and polymer.^34,35^ Here, thermal annealing of the fibers induced phase separation between MAPbI_3_ precursors and the PVP binder, the former of which diffuses to the fiber surface and crystallizes. Regions between protruding crystals remained smooth throughout annealing, likely corresponding to PVP-rich areas.

PMMA-confined films were fabricated by spin coating PMMA from toluene onto fiber films prior to annealing (Scheme 1, bottom). Figure 1c displays a cross-sectional SEM image of a PMMA-confined fiber film. PMMA was present throughout the entire depth of the film, completely filling open spaces to encapsulate the fibers. A 500 nm PMMA capping layer also was observed at the top of the film. The film height of PMMA-confined fibers was only 8 μm, indicating that PMMA compressed the fiber films from their original thickness of 17 μm. SEM images of electrospun fibers before and after spin coating neat toluene, an antisolvent for MAPbI_3_,^36^ confirmed that toluene does not dissolve the fibers or affect the fiber morphology (Figure S1a,b).

Upon annealing at 100 °C, PMMA-confined films also transitioned from pale yellow to dark brown. To image the fiber morphology after annealing, PMMA was selectively removed by immersing the samples in chlorobenzene, an antisolvent for MAPbI_3_, for 30 min. SEM images of annealed fibers before and after immersion in chlorobenzene confirmed that chlorobenzene does not affect the fiber morphology (Figure S1c,d). Figure 1d displays top-view SEM images of PMMA-confined films annealed at 100 °C for 0, 20, 40, and 60 min. These fibers appeared smooth at all annealing times. Small, densely packed crystallites formed along the fiber but did not protrude from the fiber surfaces. These results indicate that PMMA suppresses the phase separation and blooming of MAPbI_3_, limiting crystal growth to along the fiber surface.

Figure 1e displays 1D X-ray diffraction patterns collected in air at room temperature on unconfined (top) and PMMA-confined (bottom) MAPbI_3_ fibers for annealing times between 0 and 60 min in a N_2_-filled glovebox. The simulated powder pattern of β-MAPbI_3_^37^ is displayed in black. Diffraction peaks corresponding to β-MAPbI_3_ were present for all the patterns of unconfined films, even for unannealed films. In a previous report, lattice strain was observed with CsPbI_3_ crystals nanoconfined in anodized aluminum oxide scaffolds due to an order of magnitude difference in their thermal expansion coefficients.^27^ Here, we do not observe lattice strain in the XRD patterns of nanoconfined MAPbI_3_ crystals due to the similar thermal expansion coefficients of 15.7 × 10^–5^/K for MAPbI_3_^38^ and 7.1 × 10^–5^/K for PMMA.^39^ We noted a color change of the unannealed film from yellow to gray/brown upon initial exposure to air, suggesting that oxygen or moisture induced some MAPbI_3_ crystallization without the need for thermal annealing.^40−43^ These XRD peaks increased in intensity with increasing annealing time, consistent with increased conversion to MAPbI_3_ that was observed in SEM. All patterns exhibited a broad amorphous peak around 2 Å^–1^, corresponding to the PVP binder within the fibers (Figure S2).

The XRD pattern collected on an unannealed, PMMA-confined film did not exhibit diffraction peaks associated with MAPbI_3_. Instead, weak diffraction peaks were indexed to a PbI_2_/DMF complex previously reported in the literature, (MA)_2_(DMF)Pb_3_I_8_.^44^ We also did not observe any color change upon exposing the PMMA-confined film to air, suggesting that PMMA both hinders DMF evaporation and acts as a barrier against oxygen and moisture. The ability of PMMA to protect metal halide perovskites from oxygen and moisture previously has been attributed to its hydrophobic nature and surface passivation.^45−48^

Upon annealing, peaks associated with the solvent complex phase disappeared, while peaks corresponding to β-MAPbI_3_ emerged and intensified with extended annealing times. Overall, peak intensities for XRD patterns of PMMA-confined films were lower than those of unconfined films, indicating that PMMA encapsulation partially suppresses the continued growth of MAPbI_3_ crystals. Diffraction peaks collected on PMMA-confined MAPbI_3_ films were also significantly broader, consistent with smaller crystallite sizes compared to unconfined films.

1D X-ray diffraction patterns of 0.824 M MAPbI_3_ in DMF fibers electrospun with 9, 12, and 15 wt % PVP relative to MAPbI_3_ also were collected (Figure S3a). Figure S3b displays the intensity of the MAPbI_3_ (110) diffraction peak versus PVP concentration. The intensity of diffraction peaks associated with MAPbI_3_ crystals decreases with increasing PVP content, indicating that the presence of PVP impedes MAPbI_3_ crystallization. Corresponding SEM images confirm decreasing size and density of MAPbI_3_ crystals on fiber surfaces with increasing PVP concentration. The PVP melting point of >300 °C, measured by melting point analysis of the pure PVP powder, is well above the fiber annealing temperature of 100 °C, so we expect PVP to be solid during MAPbI_3_ crystallization. MAPbI_3_ crystals thus are forced to primarily grow outward from the fiber surfaces or along the fiber surface for unconfined and PMMA-confined crystals, respectively.

**Figure 2:**
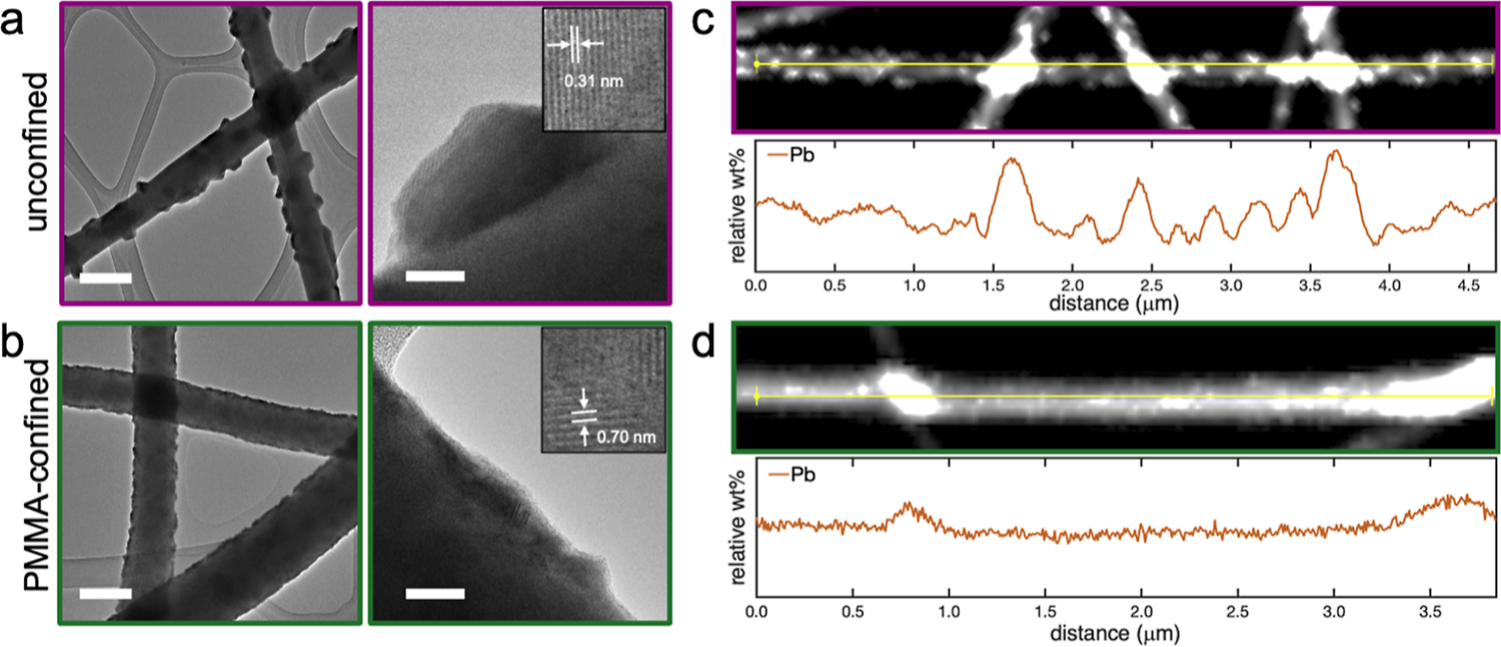
TEM images of (a) unconfined and (b) PMMA-confined MAPbI_3_ fiber films at low (left) and high (right) magnification. Scale bar: 200 nm (left), 20 nm (right). Insets highlight regions in which lattice fringes are apparent. EDS line scan showing relative wt % of Pb along (c) an unconfined and (d) a PMMA-confined MAPbI_3_ fiber.

Figure 2a displays transmission electron microscopy (TEM) images of unconfined fibers at low (left) and high (right) magnification. Crystallites protruding from smooth fiber surfaces were observed, with crystal sizes averaging ∼70 nm in diameter. At high magnification, lattice fringes of the MAPbI_3_ crystal were visible, as seen on the right of Figure 2a. For unconfined films, lattice fringes were measured to be 0.31 nm, corresponding to the (220) plane of β-MAPbI_3_ crystals.

Figure 2b displays TEM images collected on PMMA-confined MAPbI_3_ fibers that were annealed in the presence of PMMA. TEM images were collected after PMMA removal. Crystals were observed along the fiber surface but with significantly smaller sizes (∼10 nm) and higher density compared to unconfined films. At high magnification, lattice fringes were measured to be 0.33 and 0.70 nm, corresponding with the (220) and (110) planes, respectively. Lattice fringe calculations are provided in Figure S4.

It previously was reported that PMMA enables rapid heterogeneous nucleation of MAPbI_3_.^49,50^ In the PMMA-confined sample examined here, we hypothesize that heterogeneous nucleation at the PMMA/fiber interface dominates the crystallization process, resulting in many nanocrystals forming at the interface. At the same time, PMMA acts as a physical barrier to suppress crystal blooming from the fiber surface, resulting in lower overall crystallinity compared to unconfined fibers. Additionally, PVP has been used to promote MAPbI_3_ crystallization by retarding PbI_2_ formation, resulting in larger, more uniform MAPbI_3_ crystals with fewer pinholes.^51^

Figure 2c and d display electron dispersive X-ray spectroscopy (EDS) line scans of relative weight percentages (wt %) of lead (Pb) along an unconfined fiber and a PMMA-confined MAPbI_3_ fiber after PMMA removal, respectively. The unconfined fiber displays significant variations of Pb wt % along its length, with the minimum and maximum concentrations of 3% and 13%. This variation in Pb along the fiber confirms that PVP and MAPbI_3_ phase separate during thermal annealing. Not all the MAI and PbI_2_ precursors react to form MAPbI_3_, as indicated by unreacted amorphous precursor material within the noncrystalline regions of the fiber matrix. Figure S5a,c also displays EDS maps of Pb and I content across MAPbI_3_ fibers, indicating the presence of unreacted precursor material in the fibers. In contrast, the PMMA-confined fiber exhibits uniform Pb concentration, with minimum and maximum concentrations of 9% and 12%. The highest concentrations were observed at fiber intersections where fibers overlap (Figure S5b,d). The uniform concentration of Pb along the PMMA-confined fiber indicates distributed MAPbI_3_ crystallization at the PMMA/fiber interface. This data further supports that crystal blooming and MAPbI_3_/PVP phase separation is suppressed due to the presence of PMMA.

**Figure 3:**
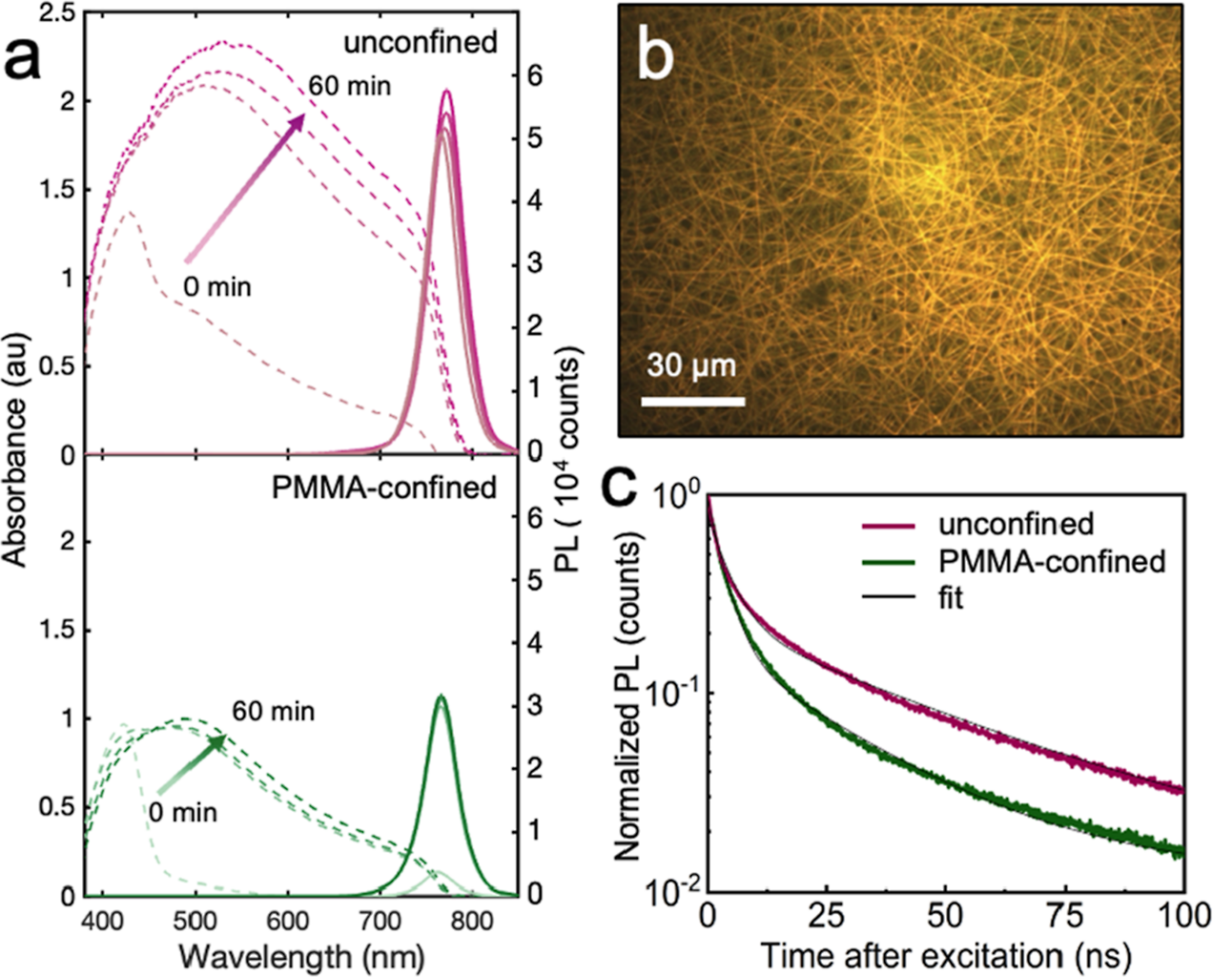
(a) Absorbance (dotted) and PL (solid) spectra of unconfined (top) and PMMA-confined (bottom) fiber films at 0, 20, 40, and 60 min of annealing. (b) Fluorescence micrograph of a MAPbI_3_ fiber film annealed for 60 min (λ_ex_ = 546 nm). (c) Time-resolved PL spectra of an unconfined fiber film and PMMA-confined film collected at λ_em_ = 767 and 762 nm, respectively (λ_ex_ = 368 nm).

Figure 3a (top) displays the absorbance and PL spectra of unconfined fiber films annealed for 0 to 60 min collected using a CRAIC spectrophotometer. The absorption spectrum of the unannealed film exhibited a peak at 425 nm followed by a broad tail from 425 to 792 nm. The peak around 425 nm previously has been attributed to tetraiodoplumbate (PbI_4_^2–^), which forms in the presence of DMF.^52,53^ The broad absorption between 425 to 792 nm indicates some conversion to the perovskite phase before thermal annealing, consistent with the XRD patterns. The absorption intensity increased across wavelengths after thermal annealing for 20 to 60 min. The absorption cutoff was 792 nm, which agrees with previous literature reports.^54−57^

PL spectra collected on the unconfined films using an excitation wavelength of 546 nm exhibited a single Gaussian peak centered at 765, 770, and 771 nm at annealing times of 0, 20, and 40 min or longer, respectively. Bulk β-MAPbI_3_ crystals emit at 820 nm.^58,59^ The shorter emission wavelengths we observed are typical for electrospun MAPbI_3_ crystals, likely due to the presence of smaller crystals that exhibit blue-shifted PL emission compared to the bulk.^31,32,60^ The increase in absorption and PL intensity with longer annealing time indicates increased conversion to MAPbI_3_, also consistent with time-dependent XRD patterns (Figure 1e). Figure 3b displays a fluorescence micrograph (λ_ex_ = 546 nm) of an unconfined film annealed for 60 min in which uniform fluorescence is observed throughout the electrospun fibers.

Figure 3a (bottom) displays the same data collected on PMMA-confined films. Spectra collected on a representative film before and after PMMA removal showed no change (Figure S6a), so PMMA was not removed for these measurements. The absorption spectrum of an unannealed PMMA-confined film displayed a main absorption peak around 418 nm, corresponding to PbI_4_^2–^. A low absorption shoulder was observed between 400 and 550 nm, indicating little to no conversion to MAPbI_3_ prior to annealing. The lack of conversion to MAPbI_3_ prior to thermal annealing is likely due to hindered DMF evaporation from the film, consistent with XRD measurements (Figure 1e), and slower oxygen and water diffusion to the fibers in the presence of PMMA.^61−64^ Upon annealing for 20 min, the absorption band broadened to 767 nm, with slightly increasing intensities for longer annealing times. Absorption spectra for PMMA-confined films exhibited lower overall intensity compared to unconfined films, indicating lower perovskite crystallinity due to the suppression of crystallization during annealing in the presence of PMMA.

Unannealed PMMA-confined fibers exhibited a low-intensity PL peak centered at 763 nm. The peak intensity reached a maximum value after 20 min of annealing and exhibited a small red shift to 766 nm. PL intensities were approximately half the values for those of unconfined films, again indicating less MAPbI_3_ formation in PMMA-confined films, consistent with time-dependent XRD patterns (Figure 1e). The PL peak for PMMA-confined fibers was also blue-shifted compared to unconfined fibers (763 nm compared to 792 nm). The blue shift of the main PL peak is attributed to the smaller crystal sizes in PMMA-confined films compared to unconfined films. Smaller crystal sizes have a wider band gap which blue shifts PL emission.^65,66^

Figure 3c displays time-resolved photoluminescence (TRPL) decay curves for an unconfined and PMMA-confined film annealed for 60 min (λ_ex_ = 368 nm). The TRPL decays were fit to a biexponential decay using the following equation
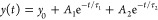
where *A*_*i*_ and τ_*i*_ are the amplitude and the lifetime of component *i*, respectively. For perovskites and other semiconductors, τ_1_ and τ_2_ are attributed to trap-mediated (fast) and radiative (slow) recombination, respectively, *A*_1_ and *A*_2_ represent the relative contributions of each process.^67^ Unconfined films exhibited τ_1_ and τ_2_ values of 4.2 and 42 ns, respectively, with amplitude contributions of *A*_1_ = 0.56 (72%) and *A*_2_ = 0.22 (28%). PMMA-confined films exhibited τ_1_ and τ_2_ values of 3.2 and 25.5 ns, respectively, with amplitude contributions of *A*_1_ = 0.77 (82%) and *A*_2_ = 0.17 (18%).

Fast decay, represented by τ_1_, dominates the decay response for both unconfined and PMMA-confined fibers, indicating that defects dominate carrier recombination.^29,30^ PMMA-confined fibers exhibited a smaller τ_1_ compared to unconfined fibers, suggesting a higher density of defects in PMMA-confined films. Bulk recombination, represented by τ_2_, is slower for unconfined crystals, consistent with previous literature reports that τ_2_ generally scales with increasing perovskite crystal size.^68^ PMMA-confined films consist of smaller crystals with larger surface-to-volume ratios, thus incorporating more surface defects and trap states. In contrast, unconfined crystals that bloom from fiber surfaces are larger, leading to a larger contribution of bulk recombination to the PL decay kinetics. These τ_1_ and τ_2_ values fall within the ranges of those previously reported for MAPbI_3_, 0.7 to 33.4 ns and 5.6 to 166.4 ns, respectively.^31,32,69^

**Figure 4:**
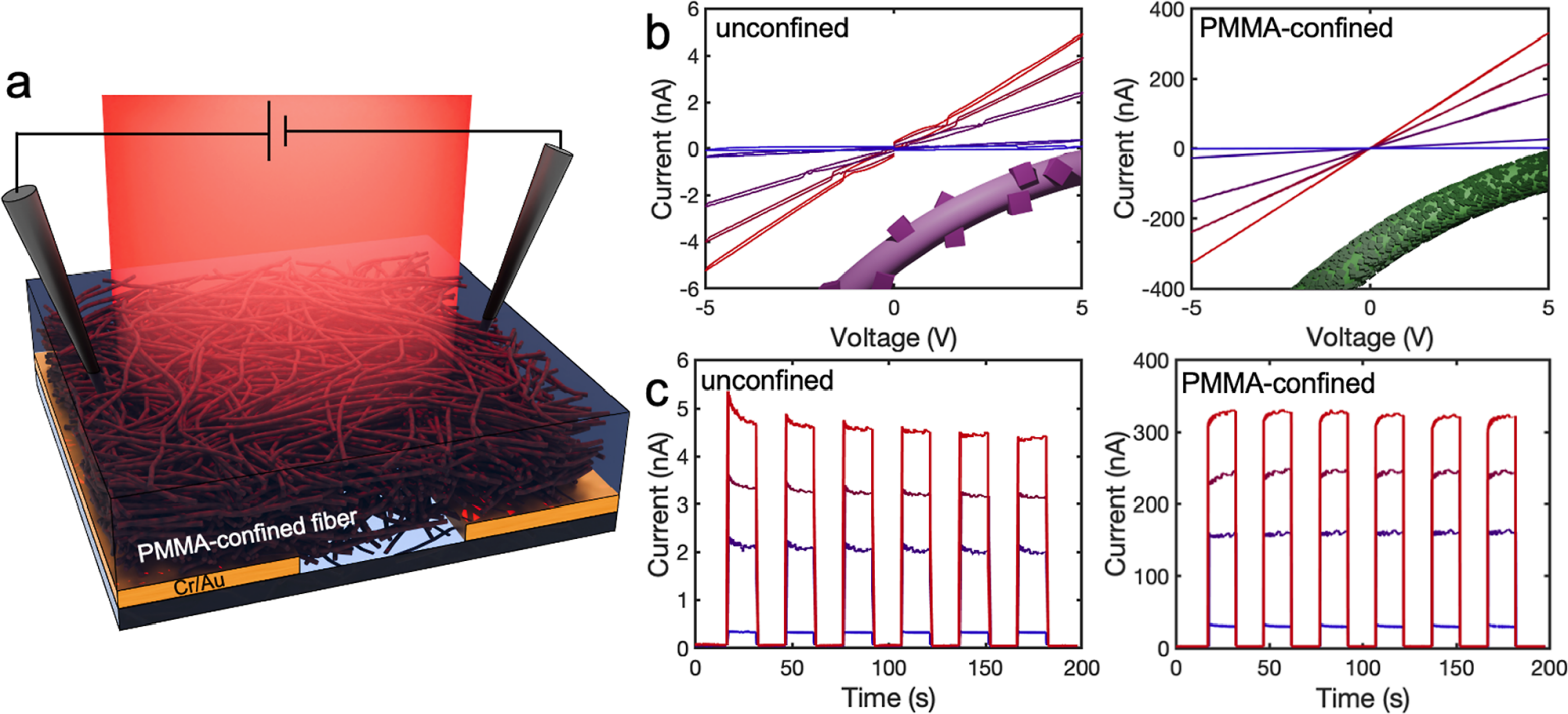
(a) Device configuration for a PMMA-confined device. (b) *I*–*V* curves and device image (inset) for a unconfined (left) and PMMA-confined (right) device varying 808 nm intensity from 0 to 48.5 mW/cm^2^. (c) Time-dependent photoresponse currents of unconfined (left) and PMMA-confined (right) with on/off switched illumination of varying the intensity of 808 nm light from 0.34 to 48.5 mW/cm^2^ at a 5 V applied bias. Insets in (b) illustrate crystal morphologies on annealed unconfined and PMMA-confined fibers.

To compare the interconnectivity between MAPbI_3_ crystals in unconfined and PMMA-confined electrospun fibers, we fabricated photodetectors with coplanar electrodes by electrospinning MAPbI_3_ precursor solutions for 12 min directly onto device platforms comprising Cr/Au coplanar electrodes deposited on glass (Figure 4a). For PMMA-confined photodetectors, PMMA was electrospun onto the fibers as previously described. Both unconfined and PMMA-confined photodetectors were then annealed for 60 min inside the glovebox. Photodetector characterization was carried out in air. Figure 4b displays the *I*–*V* curves for an unconfined (left) and PMMA-confined (right) device under 808 nm light with varying light intensity from 0 to 48.5 mW/cm^2^ at an applied bias from −5 to 5 V. The unconfined device displayed dark current and photocurrent values of 86 pA and 4.9 nA, respectively, under a light intensity of 48.5 mW/cm^2^ and a 5 V bias. Under the same conditions, the PMMA-confined device displayed dark current and photocurrent values of 1.1 and 330 nA, respectively. PMMA-confined devices consistently achieved photocurrents 2 orders of magnitude higher than those of unconfined devices across all light intensities tested.

Figure 4c displays time-dependent photoresponse currents of the unconfined (left) and PMMA-confined (right) device with intermittent 808 nm light illumination with intensities ranging from 0.34 to 48.5 mW/cm^2^ at a 5 V applied bias. At the highest light intensity, the on/off current for the unconfined device was 4.8 nA/62 pA, while the PMMA-confined device was 329 nA/2.6 nA. The rise and fall times were measured as the transition time between 10% of the minimum current to 90% of the maximum value and 90% of the maximum current to 10%, respectively. The rise and fall times for the unconfined device were 0.46 and 0.93 s, while the PMMA-confined device values were 0.08 and 0.11 s, respectively, slower than those reported for MAPbI_3_ thin film devices.^70−72^ The unconfined device displayed an initial overshoot in the photocurrent before the current stabilized. Capacitive behavior is attributed to charge accumulation within the film in the absence of percolated pathways for charges to travel to the electrodes.^73^ The PMMA-confined device displayed a small delay in current stabilization of the on-current switch, perhaps indicating the presence of some slow photocurrent carriers with inductive behavior. The slower rise time of PMMA-confined photodetectors compared to the unconfined counterpart is likely due to the higher concentration of surface traps, as revealed by TRPL measurements. Photoexcited carriers generated upon initial irradiation fill these traps instead of contributing to the device photocurrent, resulting in a transient decrease in photoconductivity.^74^ In both devices, current saturation and depletion occurred relatively quickly, with no large changes in shape or maximum current over 6 iterations of on/off switches. Additional device characterization, including responsivity and external quantum efficiency calculations, is provided in the Supporting Information.

We attribute the sixty-fold larger photocurrent of PMMA-confined devices compared to unconfined devices to the presence of a percolated network of MAPbI_3_ crystals in the former devices. MAPbI_3_ crystals grown at the PMMA/fiber interface form a dense layer of interconnected crystals through which charge carriers can easily flow (green fiber in the Figure 4b inset). In unconfined devices, on the other hand, insulating PVP-rich regions separate bloomed MAPbI_3_ crystals and act as barriers for charge transport (pink fiber in the Figure 4b inset).

PMMA encapsulation previously has been demonstrated to improve the stability of metal halide perovskites against thermal-^47,75^ and moisture-induced degradation.^76−80^ Consistent with these findings, X-ray diffraction patterns collected on unconfined and PMMA-confined MAPbI_3_ fibers stored in air for a period of 42 days demonstrated improved stability upon PMMA encapsulation. Peaks associated with MAPbI_3_ completed disappeared from the diffraction pattern of unconfined MAPbI_3_ fibers after 28 days of storage in air, while peaks associated with PbI_2_ appeared and increased in intensity over time (Figure S7a). The diffraction patterns collected on PMMA-confined fibers, on the other hand, did not significantly change in intensity over the same time period. These results indicate the PMMA can be used to both control MAPbI_3_ crystallization and improve air stability.

Figure S7b compares the photodetector performance of PMMA-confined and unconfined devices over 19 days in air. The unconfined device maintained about 70% of its initial photocurrent for the first 16 days, but by day 19 its photocurrent fell sharply to ∼10%. Concurrently, the unconfined photodetector’s color shifted from dark brown to yellow—characteristic of PbI_2_ formation that is in line with the XRD data. This observation suggests that two degradation mechanisms are occurring: one at the film surface and another at the buried film/electrode interface where charge transport occurs. At the film surface, the fibers are exposed to humidity which rapidly degrades the film.^81^ In contrast, the buried film/electrode interface is more stable, likely because it is less exposed to humidity. Slow degradation at this interface (as monitored by photodetector performance) may be due to photoinduced migration of ions during repeated device testing.^82,83^ The PMMA-confined devices showed a smaller initial drop down to about 80% after 4 days and then stabilized at roughly 75% of the initial photocurrent through day 19. We hypothesize PMMA slows humidity-induced degradation of MAPbI_3_, in agreement with previous reports,^61,80^ but does not prevent photoinduced degradation. Additionally, a spin-coated MAPbI_3_ device degraded completely within 4 days, indicating that the PVP binder in the electrospun fibers improves MAPbI_3_ stability, similarly shown in previous reports.^84−86^

## Conclusion

Nanoconfinement of metal halide perovskite crystals is being extensively explored to control crystal orientation, size and polymorphism, as well as improve their stability against humidity- and oxygen-induced degradation. Here, nanoconfinement is achieved not by embedding perovskite crystals within predefined nanopores of insulating scaffolds, but by electrospinning MAPbI_3_ precursor fibers and backfilling with a removable PMMA scaffold prior to thermal annealing. The significantly improved performance of PMMA-confined devices compared to unconfined devices is surprising considering their smaller crystal sizes, lower overall crystallinity, and higher trap density, all of which generally negatively impact charge transport. Instead, we attribute the sixty-fold larger photocurrent of PMMA-confined devices compared to unconfined devices to the presence of a percolated network of MAPbI_3_ crystals in the former devices. Utilizing electrospun fibers with retroactive PMMA-confined scaffolds thus represents a promising approach to use nanoconfinement to control crystallization while maintaining an interconnected, large surface-area network of perovskite crystals for optoelectronics. Tailoring polymer properties of the binder within the fibers and the scaffold, including melting points, thermal expansion coefficients and chemical interactions with the perovskite phase, will enable further control over nanoconfined crystallization.

## Supporting Information Available

Top view SEM of MAPbI_3_ fibers before and after spin coating with toluene and soaking in chlorobenzene; XRD patterns of electrospun PVP and spin coated PMMA on glass; XRD patterns and SEM images of films with 9, 12, and 15 wt % PVP content; TEM images, FFT images and line profiles of unconfined and PMMA-confined samples; Pb and I EDS maps of unconfined and PMMA-confined films; Absorbance, PL and photodetector performance of films before and after PMMA removal; XRD patterns and photodetector performance of aged films; EQE of unconfined and PMMA-confined devices, *I*–*V* curves and time-dependent photoresponse currents of post-PMMA sample, discussion of responsivity and external quantum efficiency (PDF)

## Abbreviations

SEM: scanning electron microscopy
XRD: X-ray diffraction
PL: photoluminescence
TEM: transmission electron microscopy
TRPL: time-resolved photoluminescence
EQE: external quantum efficiency
PVP: polyvinylpyrrolidone
PMMA: poly(methyl methacrylate).
