# Supplementary material for: Nanoconfined Metal Halide Perovskite Crystallization within Removable Polymer Scaffolds
Source: Cryst Growth Des. 2025 Apr 14;25(9):3003–12. doi: 10.1021/acs.cgd.5c00073 (PMC12063054; doi:10.1021/acs.cgd.5c00073)
Supplement: Supplementary file 1 — cg5c00073_si_001.pdf [file cg5c00073_si_001.pdf]

Supporting Information for

Nanoconfined metal halide perovskite crystallization within removable polymer scaffolds

Mia Klopfenstein,[a] Lance Emry,[a] Pulkita Jain,[b] Aida Alaei,[a] Ben Schmelmer,[a] Andrew Chou,[a] Trinanjana Mandal,[c] Min-Woo Kim,[d] Eray Aydil,[b] Tsengming Chou,[d] Stephanie S. Lee*,[a]

[a]Molecular Design Institute, Department of Chemistry, New York University, New York, NY 10003, USA

[b]Department of Chemical and Biomolecular Engineering, Tandon School of Engineering, New York University, Brooklyn, NY 11201, USA

[c]Department of Chemistry, New York University, New York, NY 10003, USA

[d]Department of Semiconductor Engineering, Myongji University, Cheoin-gu, Yongin-si, Gyeonggi-do, 17058, Korea

[e]Department of Chemical Engineering and Materials Science, Stevens Institute of Technology, Hoboken, NJ 07030, USA

Treatment of the films with toluene and chlorobenzene does not significantly affect the fiber integrity or the $MAPbI_3$ crystals, as displayed in Figure S1a-d.

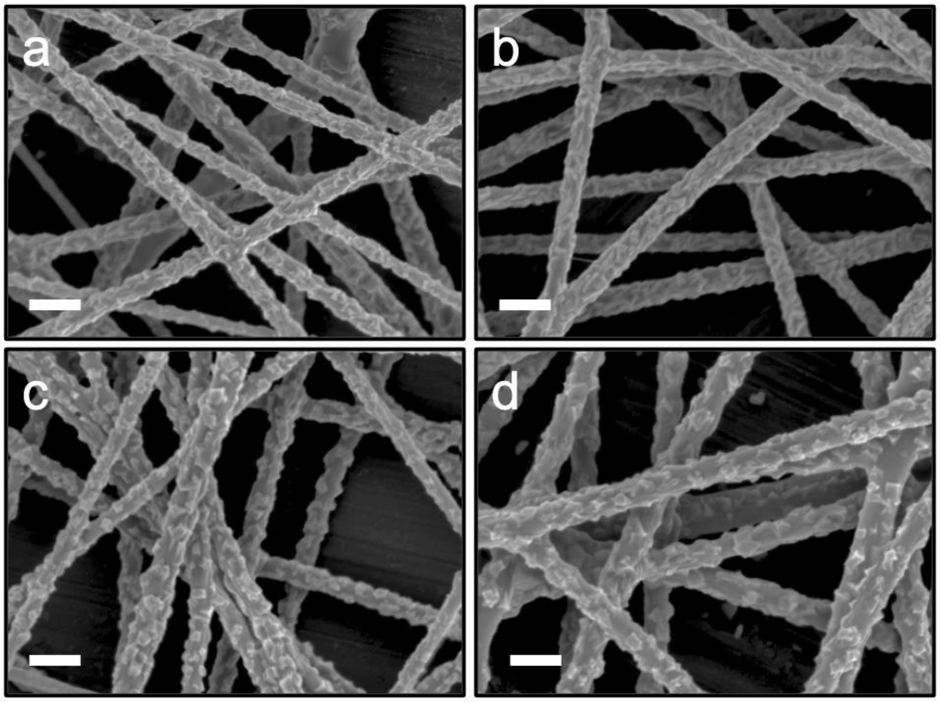

Figure S1. Top view SEM of unannealed $MAPbI_3$ fibers a) before and b) after spin coating with toluene. Top view SEM of $MAPbI_3$ fibers annealed for 60 min c) before and d) after soaking in chlorobenzene for 30 min. Scale bar = 500 nm.

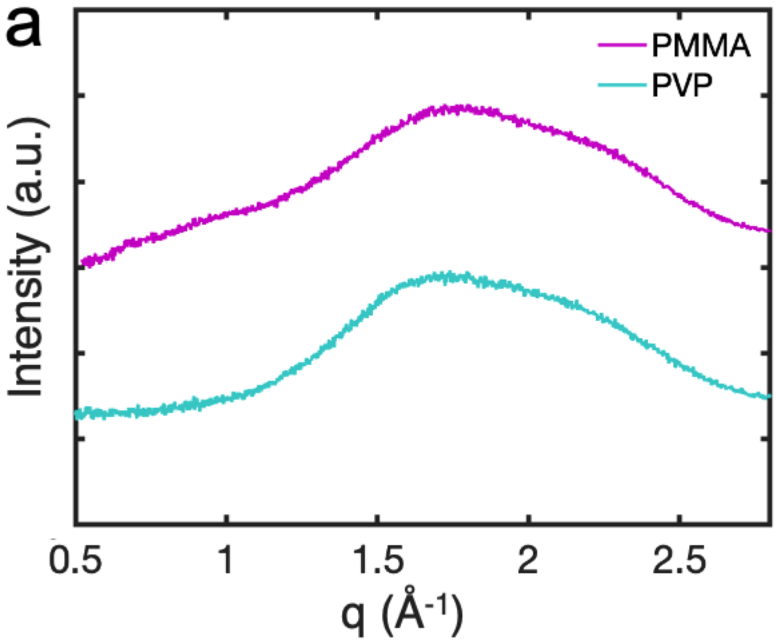

Figure S2. XRD patterns of electrospun PVP and spin-coated PMMA deposited on glass.

Figure S3 highlights the effect of PVP concentration (electrospinning binder) on the presence of $MAPbI_3$ crystals. Figure S3a displays the x-ray diffraction patterns of 0.824M $MAPbI_3$ fibers annealed at 100°C for 60 min, with increasing PVP concentrations of 9, 12 and 15 wt% (with respect to $MAPbI_3$ weight). The intensity of the diffraction peaks associated with β-$MAPbI_3$ decreases as PVP concentration increases. A closer examination of the (110) peak intensity in Figure S3b displays a significant reduction with higher PVP concentrations. Figure S3c displays SEM images of the fibers at increasing PVP concentrations, revealing that both size and density of $MAPbI_3$ crystals decrease, nearly disappearing at 15% except at fiber junctions.

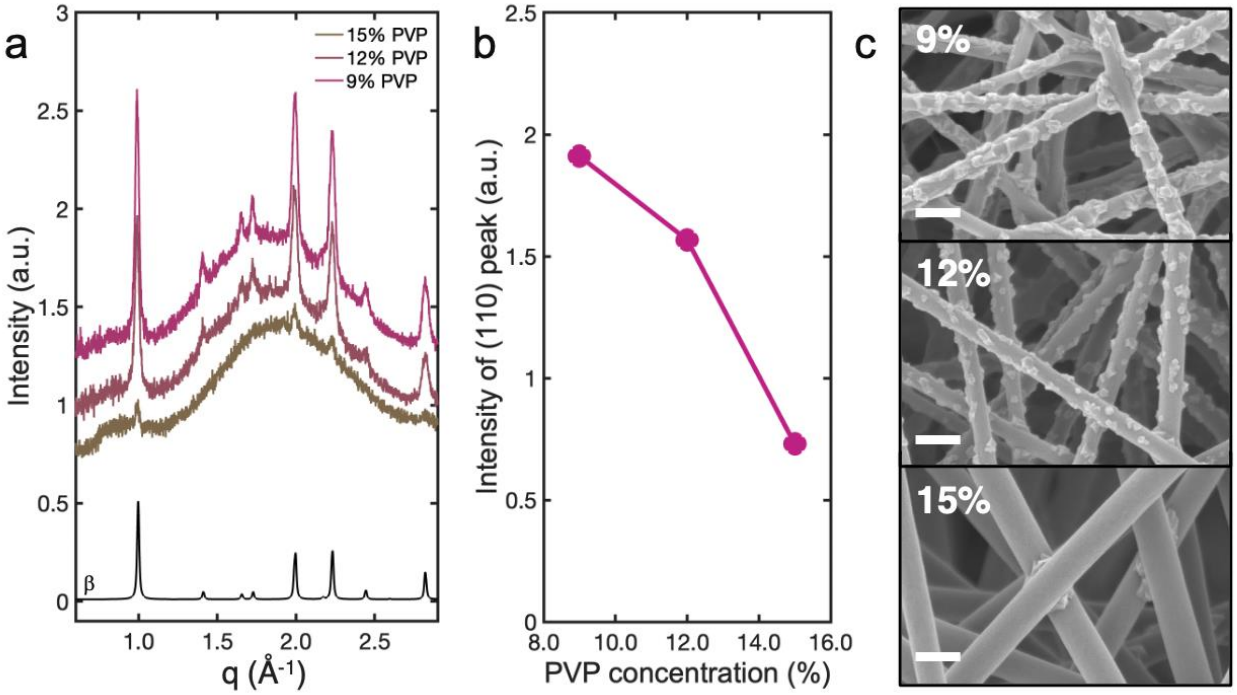

Figure S3. a) X-ray diffraction patterns b) intensity of (110) peaks of XRD patterns and c) top-down SEM images of unconfined 0.824M $MAPbI_3$ fibers with 9, 12 and 15 wt% PVP annealed at 100°C for 60 min. Scale bar = 400 nm.

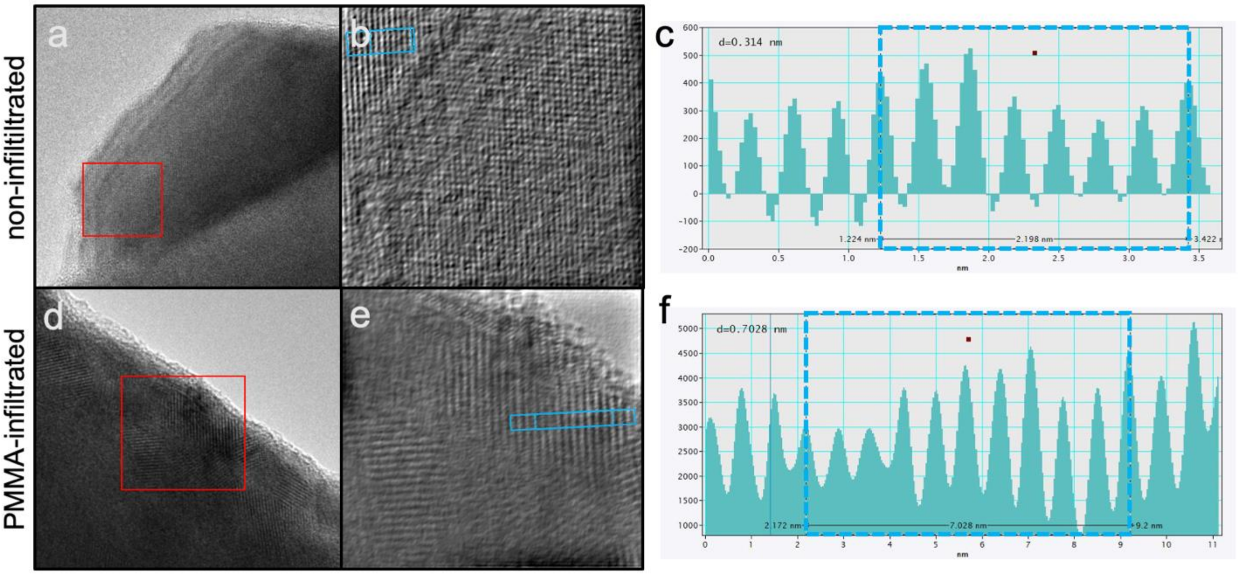

Figure S4. a) TEM image, b) image after Fourier filtering and c) line averaged intensity profile of an unconfined film. d-f) Corresponding images of PMMA-confined samples after PMMA removal.

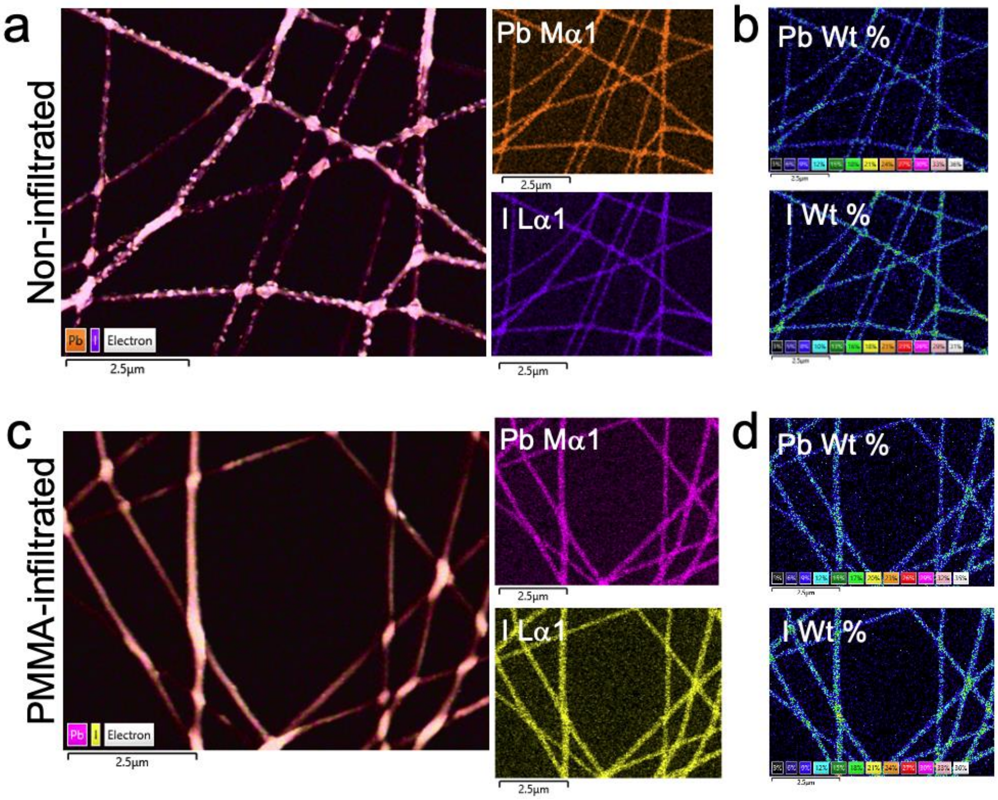

Figure S5. Pb and I EDS signals overlayed on a SEM image (left) and Pb and I EDS signal only (right) of a) unconfined and c) PMMA-confined films. Quantitative map of Pb and I wt% analyzed from EDS data of b) unconfined and d) PMMA-confined films indicating slight change in composition of Pb and I along the length of the fiber.

In Figure S6a, unconfined fibers were annealed at 100°C before spectral measurements were taken, either directly before or after the addition of PMMA. The intensity of the PL and absorbance spectra are similar, indicating that the presence of PMMA and PMMA removal have minimal impact on the intensity of the absorbance and PL spectra. In Figure S6b, photodetector measurements were taken before and after PMMA removal by soaking in chlorobenzene. The photodetector was tested under 808 nm light illumination at 48.5 mW/cm^2 intensity and a -5 to 5V applied bias. It is noted that after PMMA removal, photocurrent of the device decreased slightly.

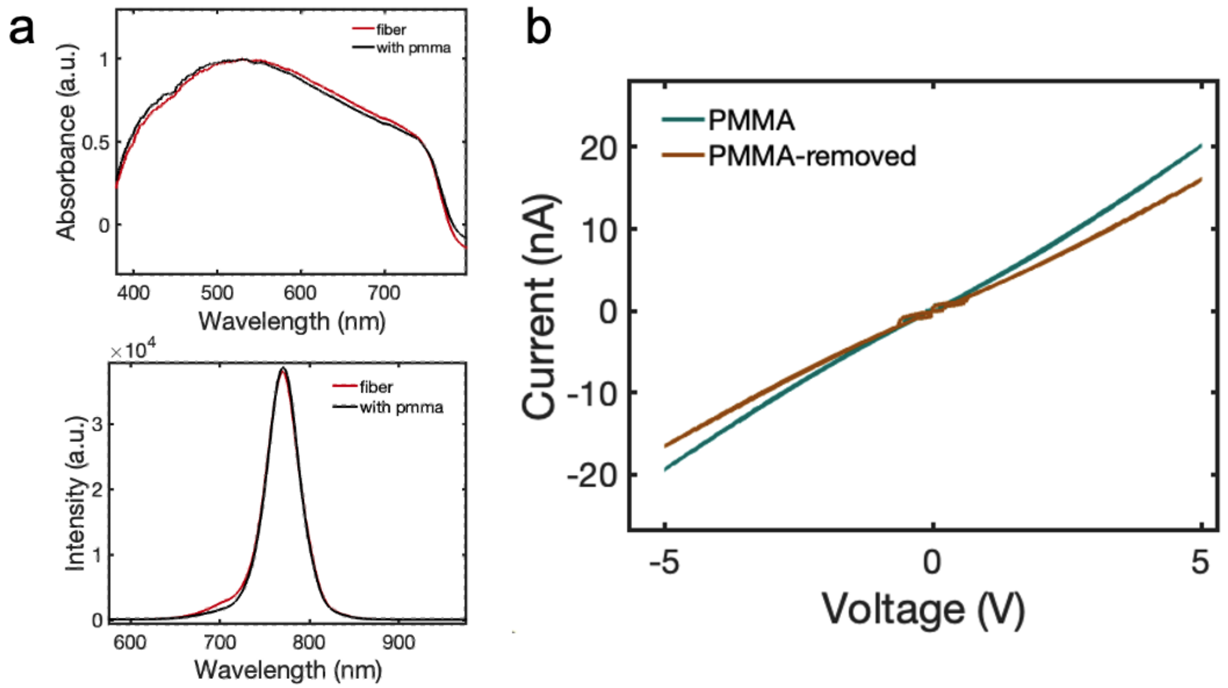

Figure S6. a) Absorbance (top) and PL (bottom) of 0.824M $MAPbI_3$ unconfined fiber annealed for 60 min at 100°C with subsequent addition of PMMA (black) or without (red). b) I-V curve of a photodetector with the same annealing conditions before and after PMMA removal under 808 nm light illumination at 48.5 mW/cm^2 intensity and a -5 to 5V applied bias**.**

X-ray diffraction patterns collected on unconfined and PMMA-confined $MAPbI_3$ fibers stored in air for a period of 42 days demonstrate improved stability upon PMMA encapsulation (Figure S7a). Peaks associated $MAPbI_3$ disappear completely from unconfined films after 28 days of storage in air. PMMA-confined films consistently showed peaks associated with $MAPbI_3$ during the 42 days. Photodetector performance of unconfined, PMMA-confined and spin coated photodetectors aged in air were tested as displayed in Figure S7b. Unconfined and PMMA-confined devices displayed significantly longer performance compared to the spin coated device owing to the PVP binder and PMMA encapsulation both preventing humidity induced degradation of $MAPbI_3$ into PbI_2. PMMA-confined films displayed the longest performance over 19 days due to the PMMA capping layer slowing the humidity-induced degradation.

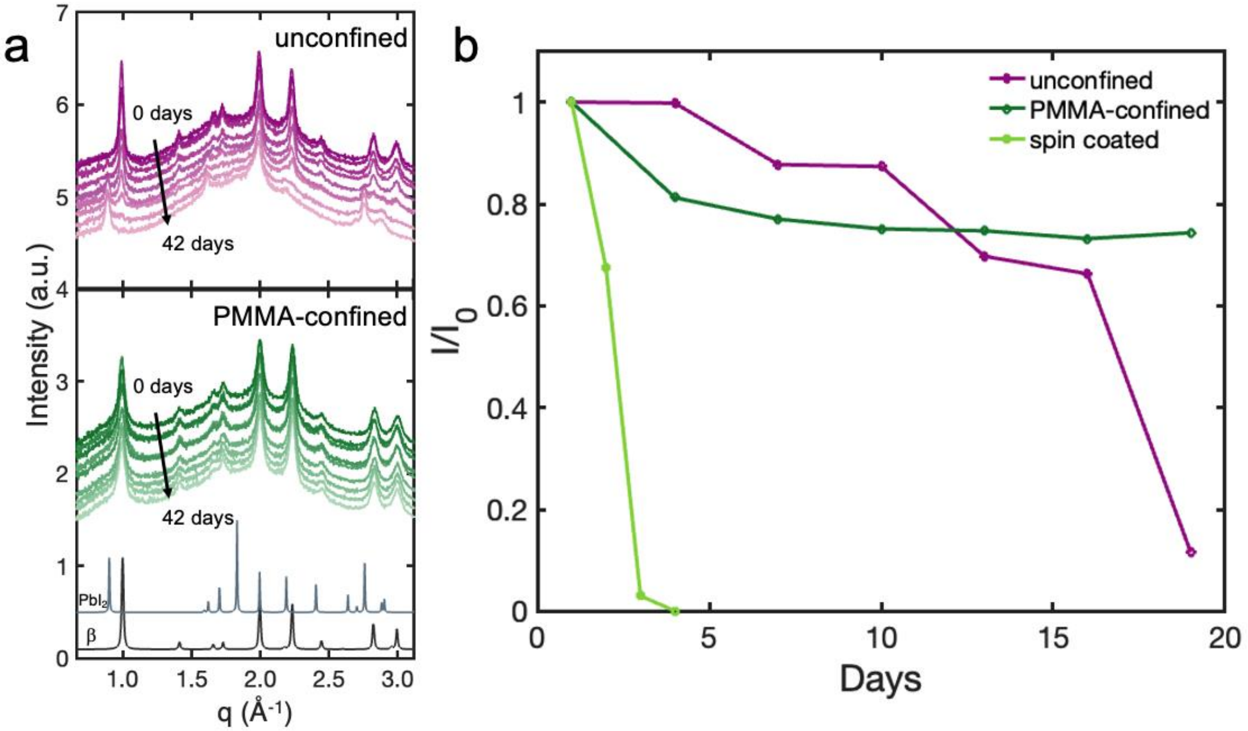

Figure S7. a) X-ray diffraction pattern of unconfined (top) and PMMA-confined (bottom) films aged in air for 42 days, with measurements taken at 1, 3, 5, 7, 10, 15, 20, 28, 42 days. b) Maximum photocurrent over 19 days of unconfined, PMMA-confined and spin coated $MAPbI_3$ photodetectors aged in air, tested under 808 nm light illumination at 48.5 mW/cm^2 at 5V.

To determine the impact of PMMA on device performance, we also fabricated a reference device for which PMMA was introduced after annealing a fiber film at 100 °C for one hour in a N2 environment and prior to testing photodetector performance in air (referred to as post-PMMA). The post-PMMA device achieved maximum dark currents and photocurrents of 51 pA and 11.6 nA, respectively (Figure S8). The maximum photocurrent is approximately double the current of the unconfined device. The addition of PMMA

compresses the electrospun fiber film, so enhanced conductivity may be attributed to increased fiber connections during compression, creating more pathways for charge transport throughout the film. Still, the post-PMMA device exhibits an order of magnitude lower current than that observed in the PMMA-confined device. These results suggest that the improved performance of PMMA-confined devices is due to the enhanced conductivity between $MAPbI_3$ crystals within fibers.

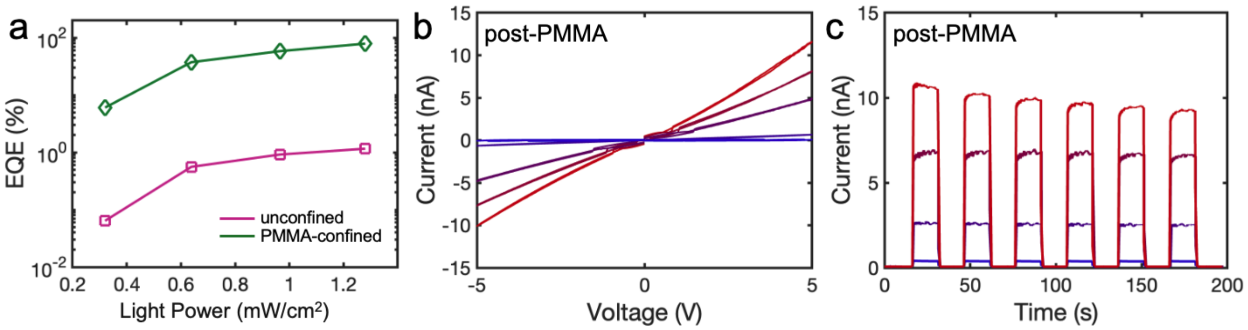

Figure S8. a) EQE at varying 808 nm light intensity from 0-48.5 mW/cm^2 for an unconfined and PMMA-confined device. Photodetector response for $MAPbI_3$ fibers annealed in the absence of PMMA and subsequently infiltrated with PMMA (both steps in a N_2-filled glove box) prior to device testing in air. b) I-V curves under 808 nm light illumination with intensities ranging from 0-48.5 mW/cm^2 and c) time-dependent photoresponse currents during intermittent irradiation with 808 nm light at intensities ranging from 0.34-48.5 mW/cm^2 at a 5 V applied bias.

Responsivity was calculated for both PMMA-confined and unconfined devices. Responsivity is defined by:

$$R_\lambda = \Delta I/PA$$

where ΔI is the difference between maximum photocurrent and dark current, P is the incident light intensity, and A is the effective area of the photodetector. Responsivity for the PMMA-confined device values was 0.013 A/W, at 5 V, 808 nm and 48.5 mW/cm^2. Responsivity values of PMMA-confined devices were 65 times higher than those of unconfined devices.

The external quantum efficiency (EQE), the ratio of charge carriers measured per number of incident photons, was calculated by:

$$EQE = \frac{R_\lambda hc}{\lambda e}$$

where h is Planck's constant, c is the speed of light, λ is the wavelength of light and e is the electronic charge. Figure S8a displays the EQE values for unconfined and PMMA-confined devices with respect to

maximum photocurrent at varying light intensities. The unconfined device displayed a maximum EQE of 0.03%, while the PMMA-confined device displayed a maximum EQE of 2.1% when irradiated with 808 nm light with an intensity of 48.5 mW/cm^2.
